# In vitro antioxidant properties, free radicals scavenging activities of extracts and polyphenol composition of a non-timber forest product used as spice: *Monodora myristica*

**DOI:** 10.1186/s40659-015-0003-1

**Published:** 2015-03-14

**Authors:** Bruno Moukette Moukette, Constant Anatole Pieme, Jacques Romain Njimou, Cabral Prosper Nya Biapa, Bravi Marco, Jeanne Yonkeu Ngogang

**Affiliations:** Laboratory of Biochemistry, Department of Biochemistry and Physiological Sciences; Faculty of Medicine and Biomedical Sciences, University of Yaoundé I, PO Box 1364, Yaounde, Cameroon; Department of Inorganic Chemistry, Faculty of Sciences, University of Yaoundé I, P.O Box 812, Yaoundé, Cameroon; Laboratory of Medicinal plant Biochemistry, Food Science and Nutrition, Department of Biochemistry, Faculty of Science, University of Dschang, PO Box: 67 Dschang, Cameroon; Department of Chemical Materials Environmental Engineering, Via Eudossiana 18, University of Rome “La Sapienza”, Rome, Italy

**Keywords:** Antioxidant, Radical scavenging, HPLC, Monodora myristica, Non-timber forest product

## Abstract

**Background:**

Excessive production of free radicals causes direct damage to biological molecules such as DNA, proteins, lipids, carbohydrates leading to tumor development and progression. Natural antioxidant molecules from phytochemicals of plant origin may directly inhibit either their production or limit their propagation or destroy them to protect the system. In the present study, *Monodora myristica* a non-timber forest product consumed in Cameroon as spice was screened for its free radical scavenging properties, antioxidant and enzymes protective activities. Its phenolic compound profile was also realized by HPLC.

**Results:**

This study demonstrated that *M. myristica* has scavenging properties against DPPH^•^, OH^•^, NO^•^, and ABTS^•^ radicals which vary in a dose depending manner. It also showed an antioxidant potential that was comparable with that of Butylated Hydroxytoluene (BHT) and vitamin C used as standard. The aqueous ethanol extract of *M. myristica* barks (AEH); showed a significantly higher content in polyphenolic compounds (21.44 ± 0.24 mg caffeic acid/g dried extract) and flavonoid (5.69 ± 0.07 quercetin equivalent mg/g of dried weight) as compared to the other studied extracts. The HPLC analysis of the barks and leaves revealed the presence of several polyphenols. The acids (3,4-OH-benzoic, caffeic, gallic, O- and P- coumaric, syringic, vanillic), alcohols (tyrosol and OH-tyrosol), theobromine, quercetin, rutin, catechine and apigenin were the identified and quantified polyphenols. All the tested extracts demonstrated a high protective potential on the superoxide dismutase (SOD), catalase and peroxidase activities.

**Conclusion:**

Finally, the different extracts from *M. myristica* and specifically the aqueous ethanol extract reveal several properties such as higher free radical scavenging properties, significant antioxidant capacities and protective potential effects on liver enzymes.

## Background

A growing human population, global climate change and the change of terrestrial food resources for energy needs in recent times have raised serious global food security concerns [1]. Further, the globalization of markets has also brought a growing globalization of foods, weakening the boundaries of human races and geographical regions of the countries throughout the world [1]. Also, there has been a quest to explore and use foods from diverse sources to enhance and supplement the nutritional quality of human foods [1]. Non timber forest products used in diet have recently gotten an increasing interest as they constitute a developing source of food. Moreover, herbs and spices derived from plants are widely used for their aromatic properties as condiments or seasonings across African countries including Cameroon and have a high impact on the food quality in this area [2,3]. Natural compounds have been reported to possess antioxidant properties, bioactivities and applications of preparations isolated from plant species, which most frequently include berries, fruits, vegetables, medicinal, aromatic plants, spices and other botanicals have been well documented [4-7]. Polyphenols are bioactive compounds broadly spread in plants and they are also significant constituents of the human diet [2]. Plants are considered as the main sources of antioxidants, which constitute a rich diversity of compounds such as flavonoids (anthocyanins, flavonols, flavones) and several classes of non-flavonoids (phenolic acids, lignins, stilbenes, terpenoids, etc.). These compounds differ in structure, the number of phenolic hydroxyl groups and their location, leading to variation in their antioxidative and biological potential [8]. Some culinary herbs and spices have been proved to be more effective antioxidants than common food additives (butylated hydroxyanisole, butylated hydroxytoluene and propyl gallate) and vitamins (ascorbic acid, α-tocopherol and β-carotene) [9]. Therefore herbs and spices rich in antioxidants and other phyto-compounds are able to prevent oxidative stress and its related disorders such as chronic diseases [10,11].

Oxidative stress originates from an imbalanced redox status between the production of reactive oxygen species (ROS) and the biological system able to remove them [12]. ROS, which include superoxide (O^2−^), hydroxyl radical (^•^OH) and hydrogen peroxide (H_2_O_2_), are continually produced in aerobic organisms. The endogenous sources of ROS are oxidative phosphorylation, P450 metabolism, peroxisomes and inflammatory cell activation [12,13]. Typically, ROS viewed as host defending molecules are released by the neutrophils for abolishing exogenous pathogens such as bacteria. They can be also formed as a result of exposure to ionizing radiation (IR), chemotherapeutic drugs and environmental contact to transition metals and chemical oxidants [14]. Cellular antioxidant defense enzymes, against oxidative stress, contain the superoxide dismutase (SOD), glutathione peroxidase (GPx) and catalase [13]. SOD and GPx present in the cytosol and mitochondria reduce the superoxide anion to hydrogen peroxide and water, and remove also the majority of hydrogen peroxide respectively [12,15]. Meanwhile, catalase, found in the peroxisomes, also eliminates high levels of hydrogen peroxide. Nonenzymatic antioxidants such as vitamin E, vitamin C, β-carotene, glutathione, and coenzyme Q function to quench ROS [16].

ROS can arbitrarily react with lipids, proteins and nucleic acids causing oxidative stress and damage in these macromolecules leading to pathogenesis of age-related and chronic diseases such as cardiovascular disease, diabetes, chronic inflammation, neurodegenerative disorders and cancers [15,17-19]. Active oxygen may be involved in carcinogenesis through two possible mechanisms: the induction of gene mutations that result from cell injury and the effects on signal transduction and transcription factors [20].

*Monodora myristica* (*M. myristica*) grows naturally in evergreen forests from Liberia to Nigeria, Cameroon, Angola and Uganda and west Kenya [21]. The fruits and seeds are dried and sold in whole or ground to be used in stews, soups, cakes and desserts. They are used as stimulants, stomachic, against headaches, sores and also as insect repellent. In the folk medicine, the bark is used in treatments of stomach-aches, febrile pains, eye diseases and haemorrhoids [21]. The exploration, characterization and application of natural antioxidants are the focus of several research teams in the Sub-Saharan Africa [4,22]. The present study aimed to (i) investigate the antioxidant properties of *M. myristica* through different *in vitro* assays, (ii) analyze the phenolic content of its leaves and bark using HPLC; and (iii) determine the protective effect of these extracts on liver enzymes.

## Results and discussion

### Free radical scavenging potential and antioxidant properties

The scavenging ability of DPPH free radical is widely used to analyze the antioxidant potential of naturally derived foods and plants. Ethanol and water-ethanol mixture were used in this study as solvents for the extraction of the low molecular weight and moderate polar substances [23]. The DPPH radical scavenging potential of the different extracts is represented in Table 1. From this Table, all the extracts showed an inhibitory potential against DPPH free radical. The inhibitory percentages vary from 25.00 ± 0.55% for the aqueous ethanol extract from the barks of *M. myristica* (AEH) to 95.25% ± 0.23% for the vitamin C. The ethanolic extract from the barks of *M. myristica* (AEE) has the highest and significant (p < 0.05) inhibitory potential among the extracts samples tested at the different concentrations compared to the other extracts. Plant acts as electron donors because of their content in phenolic compounds [24]. This may justify the DPPH radical scavenging power noted in the extracts tested. This result corroborates previous study which demonstrated that DPPH scavenging properties of plant extracts increase with the concentration of extracts [25-27].

**Table 1:** **DPPH scavenging potential of the different plant extract**

**DPPH inhibition (%)**					
**Concentrations samples (μg/mL)**	**25**	**50**	**75**	**150**	**300**
**AEE**	56.34 ± 1.34^a^	60.11 ± 2.93^a^	64.85 ± 2.21^a^	69.45 ± 3.26^a^	82.52 ± 1.46^a^
**AEH**	43.09 ± 0.82^b^	46.86 ± 0.41^b^	54.39 ± 2.51^b^	59.41 ± 2.54^b^	77.50 ± 0.63^b^
**AFE**	44.35 ± 041^b^	48.11 ± 0.40^b^	51.32 ± 0.63^b^	58.43 ± 0.54^b^	69.68 ± 2.92^c^
**AFH**	25.00 ± 0.55^c^	30.89 ± 1.76^c^	42.83 ± 1.61^c^	54.55 ± 0.28^c^	59.10 ± 0.25^d^
**Vit C**	79.21 ± 1.69^d^	85.77 ± 0.83^d^	92.74 ± 0.63^d^	93.86 ± 0.63^d^	95.25 ± 0.23^e^

Values are expressed as mean ± SD of three replicates. In the same colon the values affected with different letter (a - e) are significantly different at p < 0.05. AFE: *M. myristica* (Leaves) ethanolic extract; AEH: *M. myristica* (Barks) aqueous ethanolextract; AEE: *M. myristica* (Barks) ethanolic extract; AFH: *M. myristica* (Leaves) aqueous ethanolextract; VIT C = Vitamin C.

Several complementary methods have been proposed to assess the antioxidant activity of plant extracts and pure compounds [28]. In vitro assays for the free radical scavenging capacity are usually based on the inactivation of radicals, such as hydroxyl (OH) and nitric oxide (NO) radicals. The Table 2 presents the results of the OH radical scavenging activity of the extracts. At the lowest concentration of extract (25 μg/mL), the scavenging properties of AEE (25.03 ± 0.57%) and AFE (25.24 ± 0.91%) are higher (p < 0.05) than those of AEH (17.45 ± 0.81%) and AFH (19.33 ± 1.47%). As the concentration rises, an increase of the percentage of inhibition is observed. At the highest concentration (300 μg/mL), AEH (79.79 ± 0.56%) and AFE (77.28 ± 0.44%) showed the highest inhibition. Vitamin C used as positive control has the best scavenging activity with a percentage of inhibition of 95.77 ± 0.28%. The NO radical scavenging property of the extract is represented in Table 3. These results demonstrated that AEE (28.02 ± 1.36%) and AFH (29.71 ± 0.18%) showed the higher (p < 0.05) scavenging potential at the concentration of 25 μg/mL. The inhibitory potential of the extracts tested rise with the augmentation of concentration. At 300 μg/mL, AEE showed the highest (p < 0.05) potential 79.61 ± 0.00% followed by AFE (76.37 ± 0.26%). Vitamin C used as control showed the overall highest inhibitory potential. The mechanism involved in the scavenging activity of the samples may be attributed to the phenolic compounds found in the plant extracts as described in previous studies [15,29]. Furthermore, the radical-scavenging activity of polyphenols is relied to the molecular structure, the substitution pattern of the hydroxyl groups, the availability of phenolic hydrogen and the possibility of stabilization of the resulting HO and NO radicals via hydrogen donation or through expansion electron delocalization [8].

**Table 2:** **Hydroxyl** (**OH**) **radical scavenging potential of the different plant extracts**

**OH inhibition (%)**					
**Concentrations samples (μg/mL)**	**25**	**50**	**75**	**150**	**300**
**AEE**	25.03 ± 0.57^a^	41.96 ± 0.62^a^	57.07 ± 0.74^a^	64.80 ± 0.94^a^	76.33 ± 0.68^a^
**AEH**	17.45 ± 0.81^b^	45.11 ± 0.93^b^	57.15 ± 0.03^a^	68.95 ± 1.79^b^	79.79 ± 0.56^b^
**AFE**	25.24 ± 0.91^a^	41.00 ± 0.98^c^	52.38 ± 0.08^b^	65.49 ± 0.60^a^	77.28 ± 0.44^a^
**AFH**	19.33 ± 1.47^c^	41.96 ± 0.79^c^	48.78 ± 0.28^c^	56.25 ± 1.62^c^	71.15 ± 2.02^c^
**Vit C**	47.07 ± 0.28^d^	78.24 ± 1.48^d^	81.33 ± 0.56^d^	87.82 ± 1.46^d^	95.77 ± 0.28^d^

Values are expressed as mean ± SD of three replicates. In the same colon the values affected with different letter (a - e) are significantly different at p < 0.05. AFE: *M. myristica* (Leaves) ethanolic extract; AEH: *M. myristica* (Barks) hydroethanolic extract; AEE: *M. myristica* (Barks) ethanolic extract; AFH: *M. myristica* (Leaves) hydroethanolic extract; VIT C = Vitamin C.

**Table 3:** **Nitric oxide** (**NO**) **radical scavenging potential of the different plant extracts**

**NO inhibition (%)**					
**Concentrations samples (μg/mL)**	**25**	**50**	**75**	**150**	**300**
AEE	28.02 ± 1.36^a^	38.39 ± 2.86^a^	54.62 ± 0.09^a^	72.44 ± 0.10^a^	79.61 ± 0.00^a^
AEH	19.45 ± 0.55^b^	26.49 ± 0.12^b^	41.92 ± 1.12^b^	59.69 ± 0.34^b^	63.07 ± 0.51^b^
AFE	29.71 ± 0.18^a^	41.22 ± 0.39^a^	57.75 ± 0.18^c^	69.87 ± 1.07^c^	76.37 ± 0.26^c^
AFH	10.66 ± 0.27^d^	31.69 ± 0.14^c^	41.97 ± 1.70^b^	55.27 ± 0.96^d^	62.89 ± 2.31^b^
Vit C	35.23 ± 0.22^e^	44.03 ± 0.64^d^	69.47 ± 0.18^d^	73.26 ± 0.41^a^	85.90 ± 0.01^d^

Values are expressed as mean ± SD of three replicates. In the same colon the values affected with different letter (a - e) are significantly different at p < 0.05. AFE: *M. myristica* (Leaves) ethanolic extract; AEH: *M. myristica* (Barks) hydroethanolic extract; AEE: *M. myristica* (Barks) ethanolic extract; AFH: *M. myristica* (Leaves) hydroethanolic extract; VIT C = Vitamin C.

The ABTS method is known to be a rapid method for the determination of the antioxidant activity and could be a useful tool to screen samples and cultivars in order to obtain high content of natural antioxidants in foods [30]. The ABTS^+^ scavenging activity is presented in the Table 4. This results show that at all the concentration, the AEE showed a higher inhibitory potential compared to other samples with a maximum of the percentage of inhibition of 63.86 ± 0.05% at 300 μg/mL. Previous study suggests that the flavonoid found in the plants exerts the antioxidant action by donating of a hydrogen atom to break the free radical chain [31]. Our results demonstrated the presence of higher total phenol content (Table 5) in the AEE and AEH compared to the ethanolic extract of the leaves from *M. myristica* (AFE) and hydro-ethanolic extract of the leaves from *M. myristica* (AFH).

**Table 4:** **ABTS radical scavenging potential of the different plant extracts**

**ABTS inhibition (%)**					
**Concentrations samples (μg/mL)**	**25**	**50**	**75**	**150**	**300**
AEE	18.52 ± 0.56^a^	20.43 ± 1.30^a^	38.93 ± 0.55^a^	50.83 ± 0.61^a^	63.86 ± 0.05^a^
AEH	11.83 ± 1.25^b^	17.20 ± 0.26^b^	36.19 ± 0.62^b^	52.46 ± 0.23^a^	63.12 ± 0.45^a^
AFE	15.13 ± 0.42^c^	20.56 ± 1.02^a^	37.76 ± 0.37^a^	55.33 ± 2.18^b^	67.84 ± 0.37^b^
AFH	10.88 ± 0.26^b^	13.87 ± 0.05^c^	32.34 ± 1.29^c^	46.18 ± 1.07^c^	57.11 ± 0.38^c^
Vit C	31.22 ± 0.23^d^	37.46 ± 1.36^d^	54.75 ± 1.23^d^	70.16 ± 1.50^d^	83.99 ± 1.73^d^

Values are expressed as mean ± SD of three replicates. In the same colon the values affected with different letter (a - e) are significantly different at p < 0.05. AFE: *M. myristica* (Leaves) ethanolic extract; AEH: *M. myristica* (Barks) hydroethanolic extract; AEE: *M. myristica* (Barks) ethanolic extract; AFH: *M. myristica* (Leaves) hydroethanolic extract; VIT C = Vitamin C.

**Table 5:** **Total phenol**, **flavonoid and flavonol contents of the different plant extracts**

**Samples**	**Phytochemicals**		
	**Polyphenol(CAE/g dried extract)**	**Flavonoid(QE/g dried extract)**	**Flavonol(CAE/g dried extract)**
AEE	18.17 ± 0.51^a^	4.61 ± 0.51^a^	2.39 ± 0.69^ac^
AEH	21.44 ± 0.24^c^	5.69 ± 0.07^b^	3.63 ± 0 .00^b^
AFE	17.44 ± 0.86^ac^	3.62 ± 0.36^c^	1.62 ± 1.27^c^
AFH	16.17 ± 0.97^c^	4.73 ± 2.92^a^	2.67 ± 0.07^ab^

Values are expressed as mean ± SD of three replicates. In the same colon the values affected with different letter (a - e) are significantly different at p < 0.05. AFE: *M. myristica* (Leaves) ethanolic extract; AEH: *M. myristica* (Barks) hydroethanolic extract; AEE: *M. myristica* (Barks) ethanolic extract; AFH: *M. myristica* (Leaves) hydroethanolic extract.

**Figure 1:**
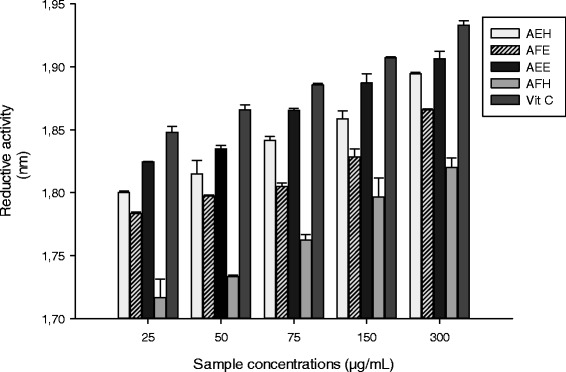
**Reductive activities of the different plant extracts.** Values are expressed as mean ± SD of three replicates. In the same colon the values affected with different letter are significantly different at p<0.05. AFE: *M. myristica* (Leaves) ethanolic extract; AEH: *M. myristica* (Barks) hydroethanolic extract; AEE: *M. myristica* (Barks) ethanolic extract; AFH: *M. myristica* (Leaves) hydroethanolic extract; VIT C = Vitamin C.

The Figure 1 represents the reductive potential of all the tested samples. The results showed that all the extracts have a reductive activity, which increase proportionally with the concentration. The samples AEE and AEH demonstrated the highest (p < 0.05) reductive potential compared to AFE and AFH but this value remain lower than vitamin C. Phenolic acids are one of the main phenolic classes within the Plant Kingdom and occur in the form of esters, glycosides or amides, but rarely in free form [32]. Within this group, flavonoids are some of the most common phenolics, widely distributed in plant tissues, and often responsible alongside the carotenoids and chlorophylls for their blue, purple, yellow, orange and red colors [32]. The total phenol, flavonoid and flavonol content of the different plants extract are represented in the Table 5. Results in this table showed that the AEH sample has the higher total phenol content (21.44 ± 0.24 mg CA/g DE) followed by AEE, AFE and AEH. Concerning the flavonoid and flavonol contents, the same extract (AEH) also showed the higher (p < 0.05) content followed by AFH, AEE and AFE. The phenolic compounds from plants are active antioxidants owing to their redox assets and chemical structure. They also have a significant role in reducing the effects of free radicals, chelating transitional metals and quenching singlet and triplet oxygen, by delocalization or decomposing peroxides [15]. The potential antioxidant activity of these phenolic compounds is demonstrated in this study through their scavenging effects on various radicals. The Table 6 presents the different values of fifty percent inhibitory concentration (IC_50_) of the tested extracts. The results demonstrated that AEE displayed the lowest IC _50_ against the DPPH, HO, NO radicals while AEH showed the lowest inhibitory potential for the ABTS radical. This results correlate the statement that the IC_50_ of the plant samples is inversely proportional to its antioxidant power thus to its phenolic content.

**Table 6:** **Different values of IC**_**50**_ **of the plant extracts on the different radicals tested**

**Samples tests**	**IC**_**50**_ **(μg/** **mL)**			
	**DPPH**	**OH**	**NO**	**ABTS**
AFE	65,10 ± 0,02^a^	157,57 ± 0,98^a^	148,28 ± 0,13 ^a^	155,16 ± 0,66 ^a^
AEH	65,80 ± 0,27^a^	99,74 ± 0,11^b^	171,38 ± 0,22 ^b^	134,17 ± 0,74 ^b^
AEE	14,66 ± 0,31^b^	28,32 ± 0,23^c^	80,07 ± 0,50 ^c^	190,06 ± 0,25 ^c^
AFH	150,66 ± 1,56^c^	154,77 ± 0,44^d^	180,18 ± 0,01 ^d^	228,43 ± 0,18 ^d^
Vit C	2,55 ± 0,46^d^	27,75 ± 0,51^e^	41,43 ± 0,58 ^e^	93,05 ± 0,07 ^e^

Values are expressed as mean ± SD of three replicates. In the same colon the values affected with different letter (a - e) are significantly different at p < 0.05. AFE: *M. myristica* (Leaves) ethanolic extract; AEH: *M. myristica* (Barks) hydroethanolic extract; AEE: *M. myristica* (Barks) ethanolic extract; AFH: *M. myristica* (Leaves) hydroethanolic extract; VIT C = Vitamin C.

The Pearson correlation analysis showed a positive correlation between the polyphenol content and the DPPH scavenging potential (Table 7). Therefore our study supports that radical scavenging power of the plant extract relies with their phenolic content [24].

**Table 7:** **Results of the Pearson correlation of the different in vitro antioxidant assays**

***TESTS***	**OH**	**NO**	**ABTS**	**DPPH**	**RED ACT**	**FLAVONOLS**	**POLYPHENOL**	**FRAP**	**MOLYBDAT**	**FLAVONOIDS**
OH	1									
NO	0,683	1								
ABTS	0,965*	0,815	1							
DPPH	0,880*	0,761	0,838	1						
RED ACT	0,811	0,715	0,766	0,983*	1					
FLAVONOLS	0,831	0,265	0,673	0,703	0,612	1				
POLYPHENOL	0,984*	0,572	0,906*	0,861	0,786	0,917*	1			
FRAP	0,633	0,143	0,433	0,667	0,604	0,920*	0,752	1		
MOLYBDAT	0,816	0,456	0,689	0,812	0,718	0,941*	0,888*	0,935*	1	
FLAVONOIDS	0,802	0,261	0,633	0,724	0,640	0,994*	0,895*	0,957*	0,962*	1

*:significant values p = 0,050 (bilateral test).

MOLYBDAT: Phosphomolybdenum test; FLavonols: Flavonol assay; Polyphenol: Polyphenol assay; Flavonoids: Flavonoid assay; NO: NO radical scavenging test; ABTS: ABTS radical scavenging test; DPPH: DPPH radical scavenging test; OH: OH radical scavenging test; RED ACT: reductive activity test.

**Figure 2:**
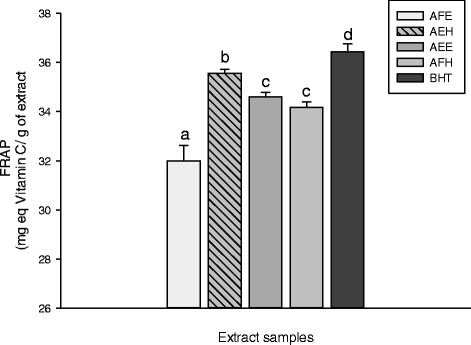
**FRAP antioxidant activities of the different plant extracts.** Values are expressed as mean ± SD of three replicates. In the same colon the values affected with different letter are significantly different at p<0.05. AFE: *M. myristica* (leaves) ethanolic extract; AEH: *M. myristica* (Barks) hydroethanolic extract; AEE: *M. myristica* (Barks) ethanolic extract; AFH: *M. myristica* (leaves) hydroethanolic extract; *BHT:Butylated hydroxyl Toluene.*

**Figure 3:**
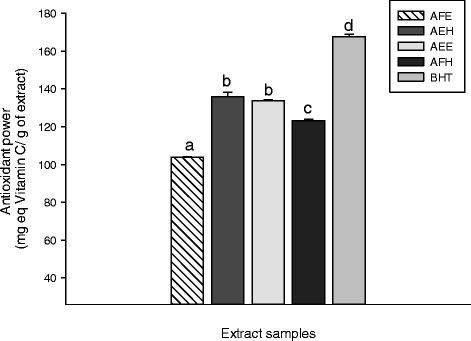
**Phosphomolybdenum antioxidant activities of the different plant extracts.** Values are expressed as mean ± SD of three replicates. In the same colon the values affected with different letter are significantly different at p<0.05. AFE: *M. myristica* (leaves) ethanolic extract; AEH: *M. myristica* (Barks) hydroethanolic extract; AEE: *M. myristica* (Barks) ethanolic extract; AFH: *M. myristica* (leaves) hydroethanolic extract; *BHT:Butylated hydroxyl Toluene*.

The use of biochemical assays to assess the antioxidant power of plants has emerged and become the best reliable and readily available methods. Because of variable response engendered by a specific antioxidant in various testing systems, it is important to utilize diverse antioxidant assays to appreciate the mechanism of action of the bioactive principle involved [33]. The FRAP and the phosphomolybdenum assays are good indicators to achieve such work. The FRAP antioxidant activities of the different tested samples are displayed in Figure 2. This result demonstrated that among these extracts, AEH exhibited the highest (p < 0.05) activity (35.57 ± 0.14 mg equivalent vitamin C/g of dried extract (mg eq VtiC/g DE)) compared to the other extracts. The Figure 3 represents the phosphomolybdenum inhibition potential of the different plant extracts. This results show that AEH (135.80 ± 2.50 mg of ascorbic acid equivalents/g of dried extract (mg eq AS/g DE)) and AEE (133.66 ± 0.73 mg eq AS/g DE) exhibited the higher (p < 0.05) antioxidant potential compared to AFH (123.15 ± 0.81 mg eq AS/g DE) and AFE (103.90 ± 0.24 mg eq AS/g DE). However these values remain lower than that of BHT (167.59 ± 1.32 mg eq AS/g DE) used as control. According to this result, we can suggest that the different phenolic compound present in our samples act as antioxidant by acting as reducing agents which convert free radicals into stable compounds [34]. These results are similar to previous studies which demonstrated the antioxidant potential of plant phytochemicals [29,35].

**Figure 4:**
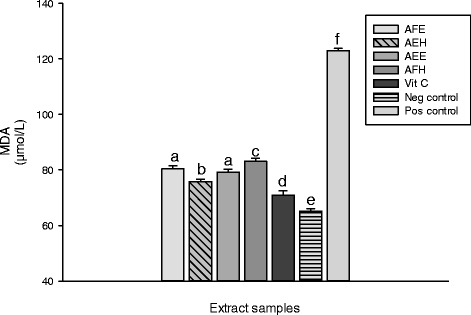
**Protective properties of plant extracts against lipid.** Values are expressed as mean ± SD of three replicates. In the same colon the values affected with different letter are significantly different at p<0.05. AFE: *M. myristica* (leaves) ethanolic extract; AEH: *M. myristica* (Barks) hydroethanolic extract; AEE: *M. myristica* (Barks) ethanolic extract; AFH: *M. myristica* (leaves) hydroethanolic extract; Vit C: Vitamin C. Pos Control: oxidant (positive) control. Neg Control: Normal (negative) control.

**Figure 5:**
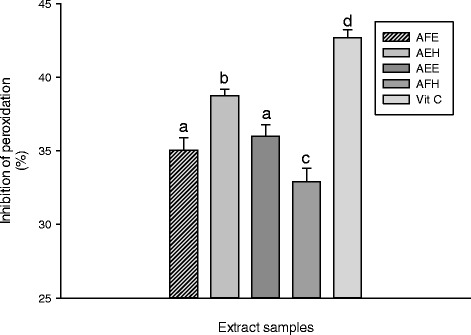
**Lipo**-**peroxidative Inhibitory potential of plant extracts.** Values are expressed as mean ± SD of three replicates. In the same colon the values affected with different letter are significantly different at p<0.05. AFE: *M. myristica* (leaves) ethanolic extract; AEH: *M. myristica* (Barks) hydroethanolic extract; AEE: *M. myristica* (Barks) ethanolic extract; AFH: *M. myristica* (leaves) hydroethanolic extract; Vit C: Vitamin C. Pos Control: oxidant (positive) control. Neg Control: Normal (negative) control.

**Figure 6:**
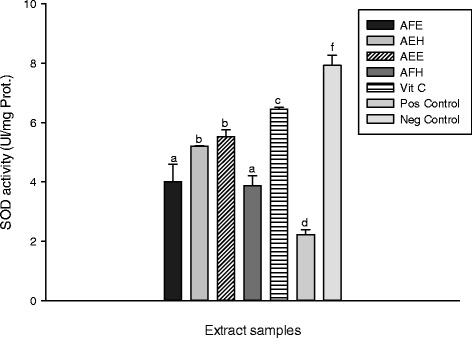
**Protective properties of plant extracts**: **SOD activity.** Values are expressed as mean ± SD of three replicates. In the same colon the values affected with different letter are significantly different at p<0.05. AFE: *M. myristica* (leaves) ethanolic extract; AEH: *M. myristica* (Barks) hydroethanolic extract; AEE: *M. myristica* (Barks) ethanolic extract; AFH: *M. myristica* (leaves) hydroethanolic extract; Vit C: Vitamin C. Pos Control: oxidant (positive) control. Neg Control: Normal (negative) control.

**Figure 7:**
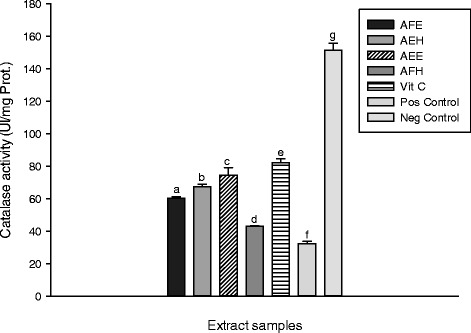
**Protective properties of plant extracts**: **catalase activity.** Values are expressed as mean ± SD of three replicates. In the same colon the values affected with different letter are significantly different at p<0.05. AFE: *M. myristica* (leaves) ethanolic extract; AEH: *M. myristica* (Barks) hydroethanolic extract; AEE: *M. myristica* (Barks) ethanolic extract; AFH: *M. myristica* (leaves) hydroethanolic extract; Vit C: Vitamin C. Pos Control: oxidant (positive) control. Neg Control: Normal (negative) control.

The inhibitory effect of the extracts from *M. myristica* on the oxidative cell damage caused by H_2_O_2_ and ^•^HO through Fenton mediated reaction was investigated by lipid peroxidation assay. In the biological system a number of end products of lipid peroxidation such as MDA constitute a significant source of cell membrane obliteration and cell injuries [36]. The results of protective potential of extracts against lipid peroxidation are represented in the Figure 4. This result showed that the oxidant (positive) control has a higher concentration of MDA (122.95 ± 0.88 μmol/L) compared to the normal (negative) control (65.13 ± 0.58 μmol/L). The vitamin C used as control has significantly (p < 0.05) inhibited the lipid peroxidation as showed the level of MDA (70.80 ± 1.46 μmol/L) and demonstrated the higher inhibitory potential (42.80 ± 0.75%) compared to the extract samples (Figure 5). Among the extract, the sample, AEH showed the lowest MDA concentration (75.82 ± 0.53 μmol/L) compared to the other samples (Figure 5). The inhibition of lipid peroxidation by antioxidant compounds is a crucial property by which they can diminish the induction and/or propagation of oxidative stress [29]. Thus, we can conclude that *M myristica* extracts have potential propriety as a protective compound against oxidative stress [36]. Enzymatic and non-enzymatic systems are used by the living organism to fight against free radical produce during oxidative stress [26,29]. The Figure 6 represents the protective effect of the plant extract on the SOD against oxidant. We can observe on this Figure that the oxidant (positive) control group exhibited the lowest SOD activity (2.22 ± 0.27 Unit/min/mg of protein (UI/mg Prot.)) compare to the other extracts including the normal group (negative) control (7.92 ± 0.13 UI/mg Prot.). Among the extract tested, AEE (5.51 ± 0.72 UI/mg Prot.) and AEF (4.05 ± 0.27 UI/mg Prot.) showed the highest protective activities. Polyphenols are well-known to possess effective scavenging activity of free radicals [2]. Therefore, phenolic compounds of *M myristica* present in the medium during the incubation time most likely quench the radicals formed in the aqueous phase before reacting with enzymes when it is later added. Our results corroborate with other studies which demonstrated that polyphenols are able to protect macromolecules from oxidative stress or increase their resistance to damage caused by oxidants [4,37]. The protective effect of the different extracts of *M. myristica* on the catalase activity is represented in the Figure 7.

**Figure 8:**
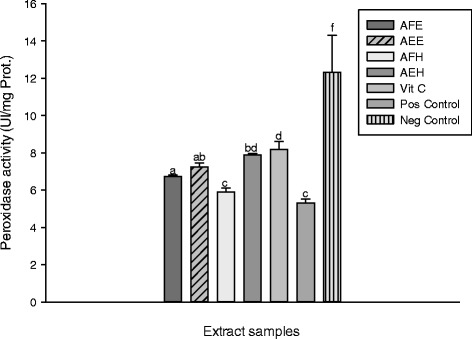
**Protective properties of plant extracts**: **peroxidase activity.** Values are expressed as mean ± SD of three replicates. In the same colon the values affected with different letter are significantly different at p<0.05. Vit C: Vitamin C; AFE: *M. myristica* (leaves) ethanolic extract; AEH: *M. myristica* (Barks) hydroethanolic extract; AEE: *M. myristica* (Barks) ethanolic extract; AFH: *M. myristica* (leaves) hydroethanolic extract.

These results showed the catalase activity is significantly decreased (p < 0.05) in the oxidant (positive) control (32.30 ± 3.95 UI/mg Prot.) compared to the other groups. Among the extract, AEE showed the highest activity (74.44 ± 1.36 UI/mg Prot.) compared to AEH (67.37 ± 0.65 UI/mg Prot.), AFE (60.34 ± 0.88 UI/mg Prot.) and AFH (43.20 ± 0.91 UI/mg Prot.). Iron contains unpaired electrons that enable them to participate in one-electron transfer reactions. Hence, they are powerful catalysts of autoxidation reactions. Thus from this results, the endogenous antioxidants of *M. myristica* may be beneficial by avoiding the deleterious effects of ion mediated oxidative stress and candidates for the prevention of oxidative damage caused by ROS [38]. The protective effects of the different samples on the peroxidase activity are represented in the Figure 8. From this Figure, we can observe that the oxidant (positive) control exhibited the lowest activity (5.15 ± .63 UI/mg Prot.) compared (p < 0.05) to the other groups including the normal (negative) control (12.30 ± 0.12 UI/mg Prot.). Among the extract samples, by AEH (7.90 ± 0.28 UI/mg Prot.) showed the highest activity followed by AEE (7.14 ± 0.31 UI/mg Prot.). The mechanism of antioxidants to remove free radicals implies the transfer of hydrogen to a free radical and hence its reduction to an unreactive species through removing the odd electron feature which is responsible for radical reactivity [38].

**Figure 9:**
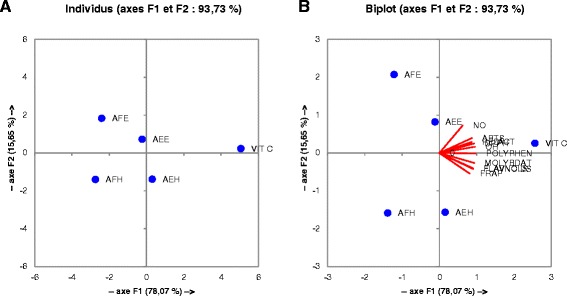
**Correlation between antioxidant capacity and free radical scavenging properties of the extracts.** AFE: *M. myristica* (leaves) ethanolic extract; AEH: *M. myristica* (Barks) hydro-ethanolic extract; AEE: *M. myristica* (Barks) ethanolic extract; AFH: *M. Miristica* (leaves) hydro-ethanolic extract; MOLYBDAT: Phosphomolybdenum test; Flavonol: Flavonol assay; Polyphenol: Polyphenol assay; Flavonoid: Flavonoid assay; NO: NO radical scavenging test; ABTS: ABTS radical scavenging test; DPPH: DPPH radical scavenging test; OH: OH radical scavenging test; RED ACT: reductive activity test; **A**: distribution of the samples around the F1 and F2 axis; **B**: projection of the samples and tests around the F1 and F2 axis.

**Figure 10:**
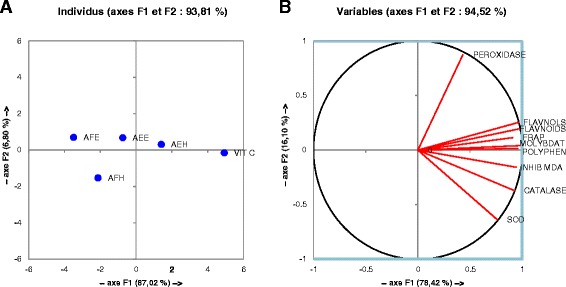
**Correlation between enzymes involved in oxidative stress and phenol contents of the extracts.** AFE: *M. myristica* (leaves) ethanolic extract; AEH: *M. myristica* (Barks) hydroethanolic extract; AEE: *M. myristica* (Barks) ethanolic extract; AFH: *M. Miristica* (leaves) hydroethanolic extract; SOD: SOD activity test; Catalase: Catalase activity test; Peroxidase: Peroxidase activity test; FLavonols: Flavonol assay; Polyphen: Polyphenol assay; Flavonoids: Flavonoid assay; FRAP: FRAP antioxidant test; MDA: MDA assay; INHIB MDA: MDA inhibition percentage; **A**: distribution of the samples around the F1 and F2 axis; **B**: projection of the samples and tests around the F1 and F2 axis.

The correlations between the free radical scavenging properties, antioxidant activity assays were also studied using the Pearson’s correlation analysis and the results are presented in the Figure 9 and Table 7. All the *M. myristica* extracts demonstrated positive and significant correlation between ABTS, OH radicals and total polyphenol with coefficient respectively of 0.984; 0.906 (Table 7). In the same way other positive significant (p < 0.05) correlations were found between phosphomolybdenum; FRAP and flavonoids or between total phenol content. These results were supported by the results of the correlation of the different extract effects on liver enzymes *in vitro* (Figure 10 and Table 8). From these results it can be noticed a positive significant (p < 0.05) (r^2^ = 0.969) correlation between the total phenol content and the lipid peroxidation inhibition. Also these results showed a positive correlation between the total phenolic content and the activities of SOD (r^2^ = 0.858), catalase (r^2^ = 0.758), peroxidase assays (r^2^ = 0.489) supporting the hypothesis that phenolic compound scavenge these free radicals by protecting therefore these macromolecules from oxidative damages. Furthermore a positive correlation has been found between the FRAP assay, Phosphomolybdenum test and SOD, catalase, peroxidase activity assays (Table 8) demonstrating that the increase of the antioxidant potential is proportionally related to the increase in the activity of antioxidant enzymes in vitro and thus a decrease in the free radicals content in the medium. To overwhelm misunderstandings concerning the choice on the most effective antioxidative extract *in vitro* and in inhibiting lipid peroxidation, and also to help reporting the most reliable antioxidant activity order of *M. Miristica* extracts based on a statistical approach, principal component analysis (PCA) was applied to the antioxidant assays data. The total phenolic flavonoid and flavonol content assays were not conducted on the pure molecules. Thus, factor analysis was performed on the data obtained only for plant extracts. A factor rotation using the Varimax method was performed for two factor loadings to see the correlations between assays that accounted for the total covariance of the plant extracts [8]. In Figure 9, the variances caused by F1 and F2 were found 78.87% and 15.65% respectively. As can be found from the PCA graph, the results from OH, DPPH, reductive activity, ABTS scavenging, Phosphomolybdenum test, flavonoid, flavonol and total phenol content assays are respectively closely loaded to F1. In contrary, the NO scavenging and FRAP assay results appear to be loaded highly close to F2. Therefore, it derived that the overall antioxidant activity of different solvent extracts of *M. Miristica* increases in the following order AFH < AFE < AEH < AEE based on the ABTS scavenging, Phosphomolybdenum test, flavonoid, flavonol and total phenol content assays values, having much higher contributions to F1 than NO assay. Similar observations are made in Figure 10 where the Phosphomolybdenum test, flavonoid, flavonol and total phenol content assay results are closely burdened to the F1 axis which has a variance of 82.07%.

**Table 8:** **Results of Pearson correlation for in vitro antioxidant assays with liver enzymes and extracts**

**Assays**	**SOD**	**CATALASE**	**PEROXIDASE**	**INHIB MDA**	**FLAVNOLS**	**POLYPHEN**	**FRAP**	**MOLYBDAT**	**FLAVNOIDS**
SOD	1								
CATALASE	0,926*	1							
PEROXIDASE	0,064	0,197	1						
INHIB MDA	0,897*	0,849*	0,315	1					
FLAVNOLS	0,793	0,565	0,631	0,872*	1				
POLYPHEN	0,858*	0,758	0,489	0,969*	0,917*	1			
FRAP	0,817	0,556	0,405	0,749	0,920*	0,752	1		
MOLYBDAT	0,906*	0,690	0,432	0,843*	0,941*	0,888*	0,935*	1	
FLAVNOIDS	0,829	0,594	0,562	0,862*	0,994*	0,895*	0,957*	0,962*	1

*: significant values p = 0,050 (bilateral test).

SOD: SOD activity test; Catalase: Catalase activity test; Peroxidase: Peroxidase activity test; FLavonols: Flavonol assay; Polyphen: Polyphenol assay; Flavonoids: Flavonoid assay; FRAP: FRAP antioxidant test; MDA: MDA assay; INHIB MDA: MDA inhibition percentage.

### HPLC phenolic profile of *M. myristica* extracts

**Figure 11:**
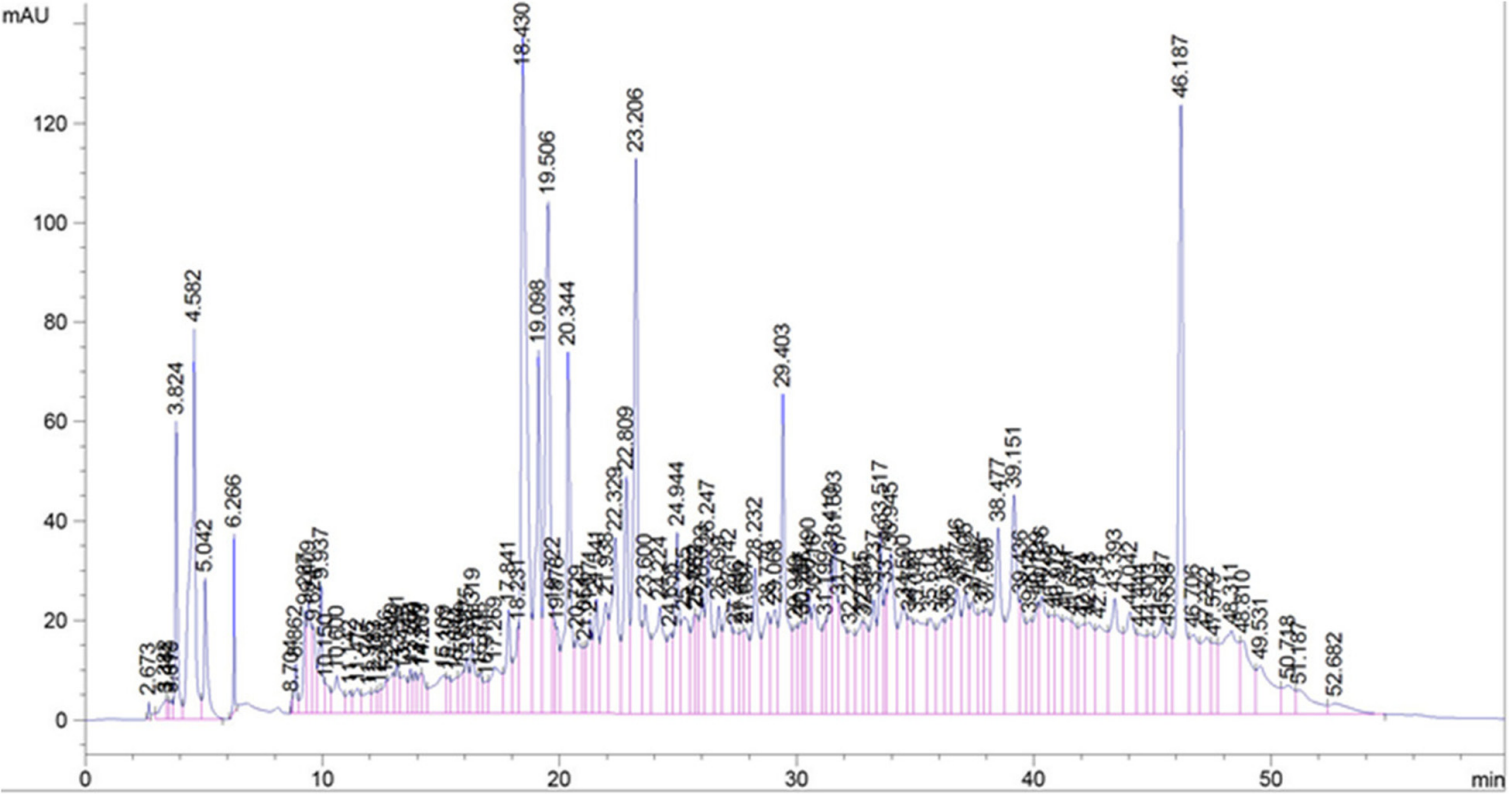
**HPLC chromatograms of phenolic extracts from the leaves of** ***M. myristica*** **recorded at 280 nm (TR: 19.10: 3,4-OH benzoic acid; 33.49:apigenin; 25.67: caffeic acid; 23.48: catechine; 29.43: eugenol; 14.38; gallic acid; 25.11: O-coumaric; 21.91:OH-tyrosol; 30.52: P-coumaric acid.** 42.19: quercetin; 29.45: rutin; 25.55: syringic acid; 17.35: theobromine; 21.77: tyrosol and 25.27: vanillic acid.).

**Figure 12:**
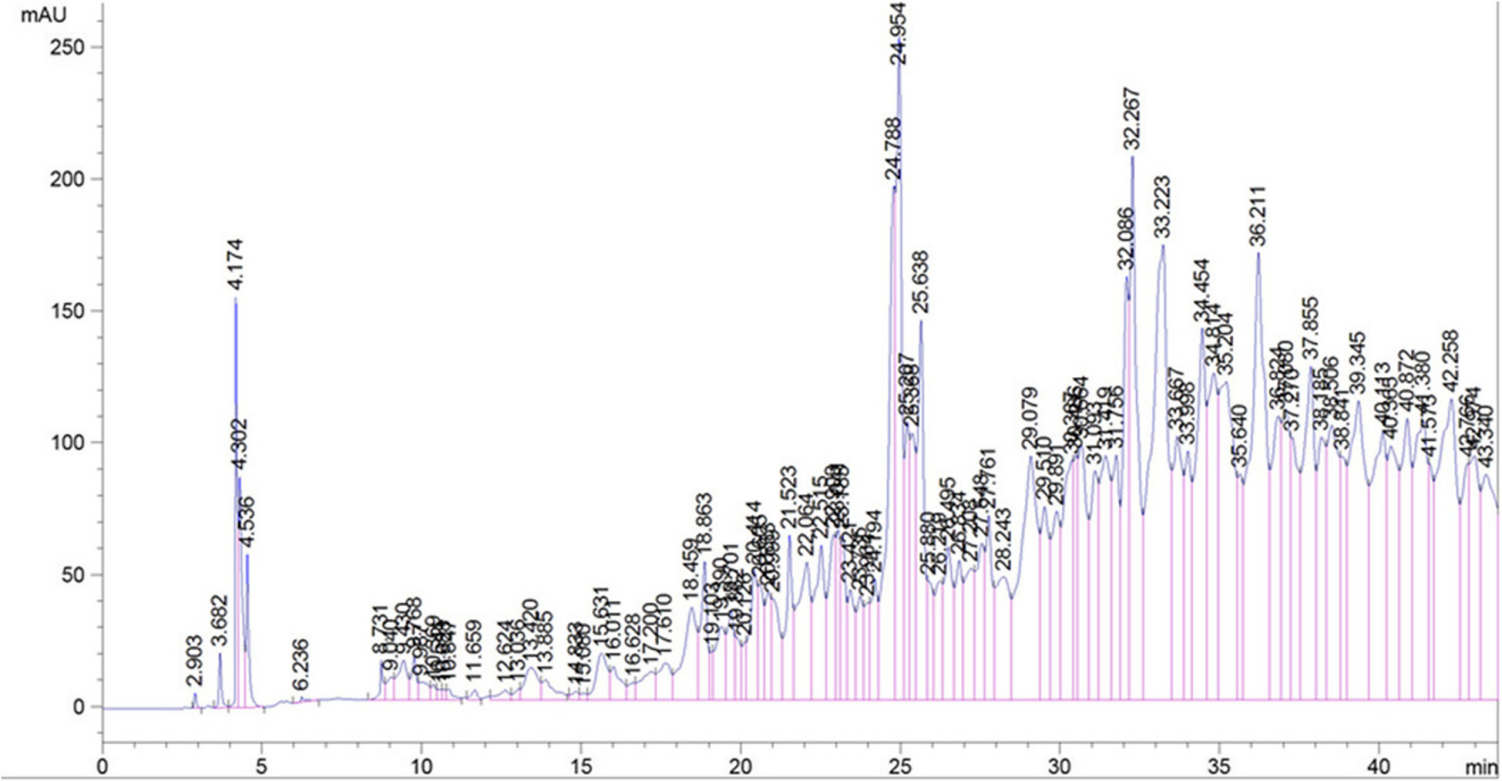
**HPLC chromatograms of phenolic extracts from the barks of** ***M. myristica*** **recorded at 280nm (TR: 19.10: 3,4-OH benzoic acid; 33.49:apigenin; 25.67: caffeic acid; 23.48: catechine; 29.43: eugenol; 14.38; gallic acid; 25.11: O-coumaric; 21.91:OH-tyrosol; 30.52: P-coumaric acid.** 42.19: quercetin; 29.45: rutin; 25.55: syringic acid; 17.35: theobromine; 21.77: tyrosol and 25.27: vanillic acid.).

Plant derived antioxidants such as polyphenols including phenolic acids, phenolic diterpenes, flavonoids, catechins, coumaric and rutin are becoming progressively more important as dietary factors. Various plants have been investigated to identify the presence of phenolic compouds using HPLC methods. The determination of phenolic compounds in the plant extracts help for their characterization and their efficient uses as important plant resources [23]. The identification of phenolic compounds in the extracts of *M. myristica* was carried out in this study. The results of the phenolic profile of the leaves and bark extracts were presented in the Figures 11 and 12 while the levels of identified phenols content are found in the Table 9. This result showed that similar phenolic compounds were presented both in the leaves and the bark of *M. myristica*, but in different concentrations. In general, the levels of these phenolic molecules were higher in the bark than the leaves (Table 9). The presence of the molecules can be classified in three groups based on the concentration. In the bark extract of *M. myristica*, the first group is made of polyphenol which concentration is higher than 100 mg/g of dry material (Quercetin, Eugenol, OH-tyrosol, Rutin), the second group (with the concentration higher than 35 mg/g of dry material) include O-coumaric acid, Tyrosol, Catechin, Caffeic acid, Vanillic acid and the last group with lower concentration of phenols (Table 9). The leaves extract of *M. myristica* showed moderate or lower concentration of phenols content. In two extracts three classes of polyphenols can also be identified. These include phenolic acids (Caffeic acid, 3,4-OH benzoic acid, Syringic acid), flavonoids (Quercetin, Catechin) and other phenolic compounds. These results corroborate with those presented in the Table 5. Total phenol value is known to reflect the overall phenolics content of a plant or food sample. Because of unidentified peaks that were not quantified in the chromatograms (Figures 11 and 12), the determination of the total phenol content seems to be incomplete. However the results of HPLC correlated and confirmed the previous results. Also this result confirmed that the classification of AEE and AEH extracts as the most powerful antioxidant among tested the extract. Phenolics are a group of non-essential dietary components and their hydrogen donating property is responsible for the inhibition of free radical induced LPO [39]. The correlation index shows that phenolics are mainly responsible for producing lipo-protective activity in the extracts. In addition other non-phenolic phytochemicals present in extracts might also be involved in imparting some degree of enzyme protection. Thus phenolic contents present in *M. myristica* extracts could be accountable for their antioxidant and protective activities. However, other studies need to be carried out to isolate individual active principles and determiner other pharmacological properties of these extracts.

**Table 9:** **Representation of the amounts of phenolic compounds in the different plant parts**

**Phenolic standards characteristics**	**Standard retention time**	**M. myristica (barks)**		**M. myristica (leaves)**	
	**T.R (min)**	**A(mUA)**	**Conc (mg/g DW)**	**A(mUA)**	**Conc (mg/g DW)**
3,4-OH benzoic acid	19,10 ± 0.00	111.81 ± 0.00	4.41 ± 0.00	940.31 ± 0.00	37.09 ± 0.00
Apigenin	33,49 ± 0.00	6 317.86 ± 0.00	1.55 ± 0.00	453.52 ± 0.00	0.11 ± 0.00
Caffeic acid	25,67 ± 0.00	2 082.25 ± 0.00	39.06 ± 0.00	158.70 ± 0.00	2.98 ± 0.00
Catechine	23,48 ± 0.00	618.04 ± 0.00	44.92 ± 0.00	1437.60 ± 0.00	104.49 ± 0.00
Eugenol	29,43 ± 0.00	1 306.65 ± 0.00	377.73 ± 0.00	365.93 ± 0.00	105.78 ± 0.00
Gallic acid	14,38 ± 0.00	49.40 ± 0.00	1.27 ± 0.00	91.85 ± 0.00	2.35 ± 0.00
O-coumaric acid	25,11 ± 0.00	3 068.61 ± 0.00	94.73 ± 0.00	486.21 ± 0.00	15.01 ± 0.00
OH-tyrosol	21,91 ± 0.00	1 404.24 ± 0.00	129.72 ± 0.00	433.30 ± 0.00	40.03 ± 0.00
P-coumaric acid	30,52 ± 0.00	1 814.51 ± 0.00	35.53 ± 0.00	518.64 ± 0.00	10.16 ± 0.00
Quercetin	42,19 ± 0.00	4 690.84 ± 0.00	440.71 ± 0.00	554.40 ± 0.00	52.09 ± 0.00
Rutin	29,45 ± 0.00	1 306.65 ± 0.00	110.01 ± 0.00	988.50 ± 0.00	83.23 ± 0.00
Syringic acid	25,55 ± 0.00	1 127.80 ± 0.00	27.96 ± 0.00	252.70 ± 0.00	6.26 ± 0.00
Theobromine	17,35 ± 0.00	329.19 ± 0.00	11.04 ± 0.00	308.09 ± 0.00	10.33 ± 0.00
Tyrosol	21,77 ± 0.00	851.15 ± 0.00	49.72 ± 0.00	323.90 ± 0.00	18.92 ± 0.00
Vanillic acid	25,27 ± 0.00	1 122.82 ± 0.00	35.87 ± 0.00	456.15 ± 0.00	14.57 ± 0.00

Conc: concentration; DW: Dried Weight; T.R: retention time; A: area.

## Conclusion

From the present study, we can conclude that *M. myristica* extracts exhibited higher free radical scavenging properties as well as the protective potential against few markers of liver homogenate involved on oxidative stress and this could be linked to their antioxidant potential capacity. This information is supported by the results of HPLC of phenolic profile of the extracts. However, further investigations need to be done either to isolate the antioxidant compounds or to determine the in vivo biological activity of these extracts.

## Methods

### Plant material

The leaves and barks of *M. myristica* were collected at the Kala Mountain in the Center region of Cameroon. They were authenticated by M. NANA, a botanist of the National Herbarium of Cameroon, in comparison to the voucher specimens (27690/SFR/CAM).

### Preparation of plant extracts

The collected leaves and barks were dried at ambient temperature and crushed. The powders were then macerated in the ratio of 1:10 (w/v) for 48 h in ethanol for the ethanolic extract and in a mixture of water + ethanol (30/70); pH = 3 for the hydro-ethanolic extract. The mixtures were then filtered using a Buchner funnel and Whatman No 1 filter paper. This process was repeated once on the residue. The filtrate was concentrated using a rotavapor and the solution was dried in the oven at 55°C for two days. Each crude extract obtained was labelled using the following codes: AFE: *M. myristica* (leaves) ethanolic extract; AEH: *M. myristica* (Barks) hydroethanolic extract; AEE: *M. myristica* (Barks) ethanolic extract; AFH: *M. myristica* (leaves) hydroethanolic extract. The different samples were then kept at 4°C. Prior to the experimentation, the solutions of the four plant extracts were dissolved using ethanol different dilutions (25, 50, 75, 150, 300 μg/mL) of each.

#### Animals

Male albino *Wistar* rats weighing 200–250 g were used in this study. The rats were maintained at room temperature under the lab conditions and were fed with standard diet and water ad libitum. Livers were collected after decapitation of the rats under mild ether anesthesia from overnight fasted rats. This study was carried out with approval from the animal Ethics Committee of university of Yaoundé I.

### Determination of the free radical scavenging potential of the samples

#### Scavenging activity of DPPH radical

The DPPH assay measures the free radical scavenging capacity of the extracts as described previously [40]. Three milliliters of each of the diluted extracts were put in the test tube and 1 mL of a methanol solution of DPPH (0.1 mM) was added. The mixture was kept in the dark at room temperature for 30 min and absorbance was measured at 517 nm against a blank. The same procedure was used for the vitamin C used as standard. The following equation was used to determine the percentage of the radical scavenging activity of each extract.

$$ Scavenging\; effect\left(\%\right)=100\times \left({A}_0-{A}_S\right)/{A}_0 $$

where Ao is the absorbance of the blank and As the absorbance of the sample.

### Scavenging effect of the ABTS+ radical

The ABTS assay was based on a previously described method [41] with slight modifications. ABTS radical cation (ABTS+) was produced by the reaction of a 7 mM ABTS solution with potassium persulphate (2.45 mM). The ABTS^+^ solution was diluted with ethanol to an absorbance of 0.70 ± 0.05 at 734 nm. The mixture was stored in the dark at room temperature for 12 h before used. After addition of 25 μL of extract sample or vitamin C used as standard to 2 mL of diluted ABTS^+^ solution, absorbance was measured at 734 nm after exactly 6 min. The decrease in absorption was used for calculating scavenging effect values. The following equation was used to determine the percentage of the radical scavenging activity of each extract.

$$ Scavenging\; effect\left(\%\right)=100\times \left({A}_0-{A}_S\right)/{A}_0 $$

where Ao is the absorbance of the blank; As is the absorbance of the sample.

### Nitric oxide scavenging activity

Nitric oxide scavenging activity was determined according previous authors [42]. The reaction mixture contained 2 mL of sodium nitroprusside (10 mM) in 0.5 mL phosphate buffer (0.5 M; pH 7.4). Various concentrations (25, 50, 75, 150, 300 μg/mL) of the extracts (0.5 mL) were added in a final volume of 3 mL. After incubation for 60 min at 37°C, Griess reagent [α-napthyl-ethylenediamine (0.1%) and sulphanilic acid (1%) in H_3_PO_4_ (5%)] was added. The pink chromophore generated during diazotization of nitrite ions with sulphanilamide and subsequent coupling with α-napthyl-ethylenediamine was measured spectrophotometrically at 540 nm. Ascorbic acid was used as a positive control. The scavenging ability (%) of the nitric oxide was calculated using the formula:

$$ Scavenging\; effect\left(\%\right)=100\times \left({A}_0-{A}_S\right)/{A}_0 $$

where Ao is the absorbance of the blank and As the absorbance of the sample.

### Hydroxyl radical scavenging activity

The scavenging activity of the extracts on hydroxyl radical was measured according to a previously described method [43]. To 1.5 mL of each diluted extract, 60 μL of FeCl_3_ (1 mM), 90 μL of 1,10-phenanthroline (1 mM), 2.4 mL of phosphate buffer (0.2 M; pH 7.8) and 150 μL of H_2_O_2_ (0.17 M) were added respectively. The mixture was then homogenized using a vortex and incubated at room temperature for 5 min. The absorbance was read at 560 nm against the blank. The percentage of the radical scavenging activity of each extract was calculated from the equation below:

$$ Scavenging\; effect\left(\%\right)=100\times \left({A}_0-{A}_S\right)/{A}_0 $$

where Ao is the absorbance of the blank and As the absorbance of the sample.

### Determination of the total antioxidant potential of the different samples

#### Total antioxidant activity by Ferric Reducing Antioxidant Power assay (FRAP)

The FRAP was determined using a previously described method [44] with slight modifications. The fresh FRAP reagent consisted of 500 mL of acetate buffer (300 mM; pH 3.6), 50 mL of 2,4,6- Tri (2-pyridyl)-s-triazin (TPTZ) (10 mM), and 50 mL of FeCl_3_•6H_2_O (50 mM). The colorimetric measurement was performed at 593 nm and the measurement was monitored up to 12 min on 75 μL of each extract and 2 mL of FRAP reagent. The vitamin C was used to draw a standard curve and the Butylated Hydroxy Toluene (BHT) was used for the comparison. The results were expressed as mg equivalent vitamin C/g of dried extract (mg eq VitC/g DE).

### Phosphomolybdenum Antioxidative Power (PAP)

The total antioxidant activity of the extracts was evaluated by green phosphomolybdenum complex [45]. An aliquot of 10 μL of the extract solution was mixed with 1 mL of reagent solution (0.6 M sulphuric acid, 28 mM sodium phosphate and 4 mM ammonium molybdate) in a micro centrifuge tube. The tubes were incubated in a dry thermal bath at 95°C for 90 min. After cooling, the absorbance of the mixture was measured at 695 nm against a blank. The vitamin C was used as reference to draw the standard curve and BHT was used for the comparison. The reducing capacities of the analysed extracts were expressed as mg of ascorbic acid equivalents/g of dried extract (mg eq AS/g DE).

### Reducing power assay

The reducing power of the extracts was determined by a method described by Oyaizu [46]. Different concentrations of extracts in 1 mL of distilled water were mixed with 2.5 mL of phosphate buffer (0.2 M, pH 6.6) and 2.5 mL of potassium ferrocyanide (1%). The mixtures were incubated at 50°C for 20 min. Aliquots 2.5 mL of trichloroacetic acid (10%) were added to the mixtures and centrifuged at 3000 rpm for 10 min. The supernatant of the solution (2.5 mL) was mixed with 2.5 mL of distilled water and 0.5 mL of FeCl_3_ (0.1%). The absorbance was measured at 700 nm.

### Determination of the phenolic content of the extracts

#### Total phenol determination

The total phenol content was determined by the Folin–Ciocalteu method [47]. The reaction mixture contained 200 μL of extract, 800 μL of freshly prepared diluted Folin Ciocalteu reagent and 2 ml of sodium carbonate (7.5%). The final mixture was diluted to 7 mL with deionized water and kept in the dark at ambient conditions for 2 h to complete the reaction. The absorbance was measured at 765 nm. Caffeic acid was used as standard and the results were expressed as mg caffeic acid/g dried extract (mg CA/g DE).

#### Determination of total flavonoid content

Total flavonoid content was determined using aluminium chloride (AlCl_3_) according to a known method [48]using quercetin as a standard. A volume of 0.1 mL of spice extract was added to 0.3 mL distilled water followed by 0.03 mL of NaNO_2_ (5%). After 5 min at 25°C, 0.03 mL of AlCl_3_ (10%) was added. After a further 5 min, the reaction mixture was mixed with 0.2 mL of 1 mM NaOH. Finally, the reaction mixture was diluted to 1 mL with water and the absorbance was measured at 510 nm. The results were expressed as quercetin equivalent mg/g of dried extract (QE/g dried ext).

#### Determination of total flavonols

Total flavonols in the plant extracts were estimated using a known method [49] with modifications. To 2.0 mL of sample, 2.0 mL of 2% of ethanolic solution of AlCl3 and 3.0 mL (50 g/L) sodium acetate solutions were added. After 2.5 h of incubation at 20°C, the absorbance was read at 440 nm. The results were expressed as quercetin equivalent (mg/g) dried extract (QE/g dried ext).

#### Quantification of phenolic compounds by HPLC

High Performance Liquid Chromatography (HPLC) with UV detection is frequently used for the separation and detection of phenolic compounds in extracts. Samples were dissolved in pure water according to the ratio (0.3 g/10 mL) and centrifuged at 4706 rpm for 10 min. The supernatant was filtered through a cellulose acetate membrane filter (0.20 μm or 0.45 μm, Schleicher & Schuell) and used for analysis. The analysis was performed on an Agilent Technologies 1200 HPLC system fitted with a SUPELCOSIL LC-18 column (length 250 mm, diameter 4.6 mm, packaging size 5 mm). The column temperature was set at 20°C. The mobile phase consisted of a mixture of an aqueous solution of acetic acid (0.5%) by volume (“A”) and acetic nitrile (“B”). Elution was performed by using 100% of A for the first 2 min of the run, 40% of A and 60% of B from 2 to 60 min. The flow rate was set equal to 1 mL/min and the injection volume was 25 microlitres. Polyphenols were detected by a UV detector (280 nm). The retention times of the identified polyphenolic compounds of interest were measured by a single standard solution at a concentration of 100 mg/L.

#### Protective properties of the plant against oxidative damage

##### Preparation of liver homogenate

The liver was isolated from 3 normal albino *Wistar* rats. The organs were weighed and 10% (w/v) homogenate was prepared in phosphate buffer (0.1 M, pH 7.4 having 0.15 M KCl) using the homogenizer at 4°C [29]. The homogenate was centrifuged at 3000 rpm for 15 min and the clear cell-free supernatant obtained was used for the study.

##### Preparation of the pro-oxidative solution

The oxidant solution was prepared, directly before its utilization by adding a solution of ferric chloride 100 mM to H_2_O_2_ 0.50% prepared in phosphate buffer (0.1 M, pH 7.4). This solution was used for the investigation of the protective assays on liver enzymes.

##### Total protein content

The total protein content of the mixture of liver was measured according to the protein kit supplier method (Human Kit-Hu102536, Boehringer Ingelheim, Germany). This result was used to express the activities of the different enzymes per gram of organs.

##### In vitro lipid peroxidation assay

Lipid peroxidation assay was performed by a formerly described protocol [50]. Phosphate buffer 0.58 mL (0.1 M; PH 7.4), 200 μL sample, 200 μL liver homogenate and 20 μL ferric chloride (100 mM) were combined to form mixture a which was placed in a shaking water bath for 1 h at 37°C. The reaction was terminated by adding 1 mL TCA (10%), TBA 1 mL (0.67%) to all the tubes which were placed in boiling water bath for 20 min. Then test tubes were shifted to crushed ice bath and were centrifuged at 3000 trs/rpm for 10 min. Absorbance of the supernatant was checked at 535 nm and was calculated as nM of MDA tissue using molar extinction coefficient of 1.56 × 10^5^ /M.cm.

##### Determination of peroxidase activity

Peroxidase activity was determined by the peroxidase kit (CAS Number 7722-84-1, Sigma Aldrich) supplier with modifications. A solution containing the mixture of 1 mL of the oxidant solution (FeCl_3_, 100 mM) and extract or vitamin C (standard) for a final concentration of 100 μg/mL was incubated for 1 h in a water bath at 37°C. An aliquot of PBS (0.1 mL), hydrogen peroxide (50 μL), and pyrogallol solution (110 μL) were added to distilled water (625 μL) that was earlier dispensed into an Eppendorf tube. The plant extract (75 μL) from the mixture was thereafter added. For the blank, the control oxidant solution and the vitamin C as standard, the same reagents were used, except the extract which was replaced by distilled water (75 μL). The reaction was mixed and incubated for at least 10 min. The solution containing 100 mM, pH 6.0 PBS (40 μL) and 0.002% (v/v) diluted liver homogenate (40 μL) were added to the blank and test mixtures respectively. These were mixed, and the increase in absorbance at 420 nm was measured at every 10 s for 3 min using a spectrophotometer (BioMate 3S UV-Visible, Thermo Scientific™Manufacturer, Wohlen, Switzerland). One unit of peroxidase was defined as the change in absorbance/ seconds/mg of protein at 420 nm using molar extinction coefficient of 12 /M.cm.

##### Determination of catalase activity

Prior to the test, a solution containing a mixture of 1 mL of total volume of the oxidant solution and extract or vitamin C (standard) for a final concentration of 100 μg/mL was incubated for 1 h in a water bath at 37°C. The catalase activity of liver homogenate was assayed as previously described with modifications [51]. An aliquot of hydrogen peroxide (0.8 mL) was dispensed into an Eppendorf tube. Phosphate buffer (1.0 mL), extracted sample/Vitamin C/oxidant solution (75 μL) and (0.002% v/v) diluted homogenate (125 μL) were added. The reaction mixture (0.5 mL) was dispensed into 5% dichromate reagent (1.0 mL) and vigorously shaken. The mixture was heated in a Clifton water bath for 10 min, and allowed to cool. The absorbance at 570 nm was taken using spectrophotometer (BioMate 3S UV-Visible, Thermo Scientific™ Manufacturer, Wohlen, Switzerland). The absorbance obtained was extrapolated from the following standard curve y = 0.0028x + 0.0132. The catalase activity was thereafter expressed as Unit/min/mg of protein (UI/mg Prot.)

5 $$ CAT(unit)/ mg\; protein\Big)=\left( Abs/ \min \times 30000\; units\right)/\left(40 cm/M\times mgprotein\right)\times df $$

where df = dilution factor, Abs = absorbance.

##### Superoxide dismutase (SOD) activity

The measurement of total SOD activity was performed according to the Misra and Fridovich method with some slight modifications [52]. The principle of this method is based on the inhibition of epinephrine autoxidation. Distilled water (0.2 mL) and 2.5 mL sodium carbonate buffer 0.05 M, pH 10.2 were added to the 0.3 mL buffered epinephrine to initiate the reaction. The absorbance at 480 nm was read for 150 s at 30 s intervals against a blank made up of 2.5 mL buffer, 0.3 mL epinephrine and 0.2 mL distilled water. The following equation allowed the calculation of the SOD activity:

6 $$ SOD\left( unit/ mgprotein\right)= SO\left( unit s/ mL \min \right)/ protein\left( mg/ mL\right)\times df $$

where df = dilution factor.

The SOD activity was there after expressed as Unit/min/mg of protein (UI/mg Prot.).

### Statistical analysis

The results were presented as mean ± SD of triplicate assays. Analyses of data was conducted using one-way ANOVA (Analysis of variance) followed by Kruskal wallis test and Dunnett’s multiple test (SPSS program version 18.0 for Windows, IBM Corporation, New York, NY, USA). The Log probit was used to determinate the IC_50_ using the software XLstat version 7 (Addinsoft, New York, NY, USA) were used to achieve the Pearson Correlation Analysis (PCA). The differences were considered as significant at *P < 0.05*.

## Abbreviations

MDA: Malonaldialdehyde
DPPH: 2,2-diphenyl-1-picrylhydrazyl 1,1-diphenyl-2-picrylhydrazyl radical
Vit C: Vitamine C
ABTS: 2,2 -Azinobis(3-ethylbenzthiazoline)-6-sulfonic acid
BHT: Butylated hydroxytoluene
RNS: Reactive nitrogen species
ROS: Reactive oxygen species
FRAP: Ferric reducing antioxidant power
MDA: Malondialdehyde
TBA: Thiobarbituric acid
H_2_O_2_: hydrogen peroxide
